# Distinct Resistomes and Microbial Communities of Soils, Wastewater Treatment Plants and Households Suggest Development of Antibiotic Resistances Due to Distinct Environmental Conditions in Each Environment

**DOI:** 10.3390/antibiotics10050514

**Published:** 2021-05-01

**Authors:** Laura Schages, Florian Wichern, Stefan Geisen, Rainer Kalscheuer, Dirk Bockmühl

**Affiliations:** 1 Faculty of Life Sciences, Rhine-Waal University of Applied Sciences, 47533 Kleve, Germany; laura.schages@outlook.de (L.S.); florian.wichern@hsrw.eu (F.W.); 2 Institute of Pharmaceutical Biology and Biotechnology, Heinrich Heine University Düsseldorf, 40225 Düsseldorf, Germany; rainer.kalscheuer@hhu.de; 3 Laboratory of Nematology, Department of Plant Sciences, Wageningen University, 6708 Wageningen, The Netherlands; stefan.geisen@wur.nl

**Keywords:** antibiotic resistance, resistome, environment, household, soil, wastewater treatment plant, transfer, metagenomics

## Abstract

The use of antibiotics in humans and animals results in a release of excess antibiotic residues into the environment through wastewaters and insufficient removal in wastewater treatment plants (WWTP), leading to increasing numbers of bacteria enriched in antibiotic resistance genes (ARG). However, the potential transfer of ARG and their host bacteria between different environments remains largely unexplored. Since many factors need to be fulfilled for a transfer between different environments, we hypothesized that antibiotic resistance (ABR) is less frequently transferred between environments in the same geographical region but rather develops and clusters in each distinct environment, leading to characteristic metagenome patterns in samples of different environments. We sampled agricultural soils, a WWTP and private households and performed metagenomic analyses to evaluate differences and potential overlaps in bacterial communities and resistomes of different environments. Wastewater revealed significantly higher richness of ARG (*n* = 40) and mobile genetic elements (*n* = 52) than soil and household samples. Bacterial communities differed between the environments and antibiotic resistance factors clustered distinctly. Overall, only few overlaps of ARG between the environments were observed, leading to the conclusion that ABR predominantly develops in individual environments as caused by environmental filtering for ARG, while a transfer between different environments is less likely.

## 1. Introduction

Although antibiotic resistance (ABR) is prevalent even in the absence of anthropogenic impacts and prior to the therapeutic use of antibiotics [1,2], the number of antibiotic-resistant bacteria has increased rapidly with the use, overuse and misuse of antibiotics in human medicine and for veterinary purposes [3,4]. This spread of ABR is a serious threat challenging not only human but also animal and environmental health. Antibiotic-resistant bacteria and antibiotic resistance genes (ARG) have been analyzed in humans, animals, food, soil, and aquatic environments and were shown to be transferred within and between the different environments [3,5,6,7,8,9,10,11]. Studies indicate that the resistome is mainly shaped by bacterial composition [12,13,14] and that similarities in taxonomic community composition lead to overlaps in mobile genetic element (MGE) and ARG prevalence [15]. Therefore, analyzing both the resistome and bacterial community of different environments is crucial to identify potential reservoirs of ABR and possible transfer routes of antibiotic-resistant bacteria. Horizontal gene transfer (HGT) between bacteria promotes genetic diversity and thus the spread of ABR [16]. Gene transfer is especially mediated by MGE such as plasmids, integrons and transposons [17,18] commonly harboring ARG, enabling vertical and horizontal gene transfer via conjugation, transformation and transduction [17,19]. Therefore, environments with a large diversity of ARG and MGE are potential sources of ABR. However, HGT requires that the bacteria inhabit or at least briefly share the same environment [20]. Antibiotic resistant bacteria and ARG are especially prevalent in the influent of wastewater treatment plants (WWTP) originating from private households, industry and clinical settings. Despite a significant decrease in ARG during biological treatment, the effluent still contains ARG and resistant bacteria [6,21]. In addition, ARG and resistant bacterial species have already been detected in ground- and drinking water [22,23], which might act as a potential transmission route to other environments, such as soil. Soil represents a natural reservoir of ABR [24,25,26], since most antibiotics originate from soil bacteria and fungi as a product to eliminate competing species [27,28]. Anthropogenic pollution via wastewater used for irrigation or sewage sludge as fertilizer plays a key role in the transmission of ABR and MGE [29,30,31,32], and thus represents an important source of ARG in soil [33]. Moreover, a transfer of bacteria harboring ARG into private households via the water supply or as part of the human/animal microbiota and vice versa via domestic wastewater of washing machines, dishwashers and drains in natural environments seems possible, but the significance of the domestic area is comparatively less well understood [34,35,36,37]. However, domestic wastewater is a major component of the WWTP influent and thus might also play a role in the dissemination of ABR. Although different environments have been identified as possible sources of ABR, their relative role in ARG transfer remains unclear. There is evidence for the transfer of ARG from environmental bacteria to human pathogens [11,19,38], which highlights the importance of identifying the role of different habitats in the spread of ABR. Therefore, identifying the most prevalent bacterial taxa is important, since these taxa might enable the transfer of ARG between the environments. Furthermore, the detection of ARG that are widespread in different environments is necessary in order to comprehend the transmission pathways of ARG and antibiotic-resistant bacteria. A metagenomic approach enables the investigation of the resistome without limitation to certain organisms. Hence, it is not surprising that in many studies, metagenome analyses focusing on distinct sources such as wastewater, soil, surface water, humans and animals were performed [12,13,39,40], revealing the widespread occurrence of ABR. In this field, households are virtually not studied and integrative cross-habitat studies to investigate potential transfer routes are rare.

Here, we performed metagenome sequencing of soil, WWTP and household samples to identify potential ABR reservoirs and to understand possible transfer routes. We hypothesized that ABR rather develops and clusters in each distinct environment due to different environmental conditions with a less frequent transfer, and thus characteristic metagenome patterns can be observed in samples originating from soil, a WWTP and households in the same geographical region. The objectives of our study were (i) to identify similarities and differences of the taxonomic distribution and antibiotic resistomes of the environments under investigation, (ii) to determine the abundance and diversity of genes encoding antibiotic resistance factors such as ARG and MGE and (iii) to elucidate if, based on similarities in micro- and resistome, transfer between the different environments can be assumed.

## 2. Results

### 2.1. Bacterial Diversity and Richness of Species, ARG and MGE

**Figure 1:**
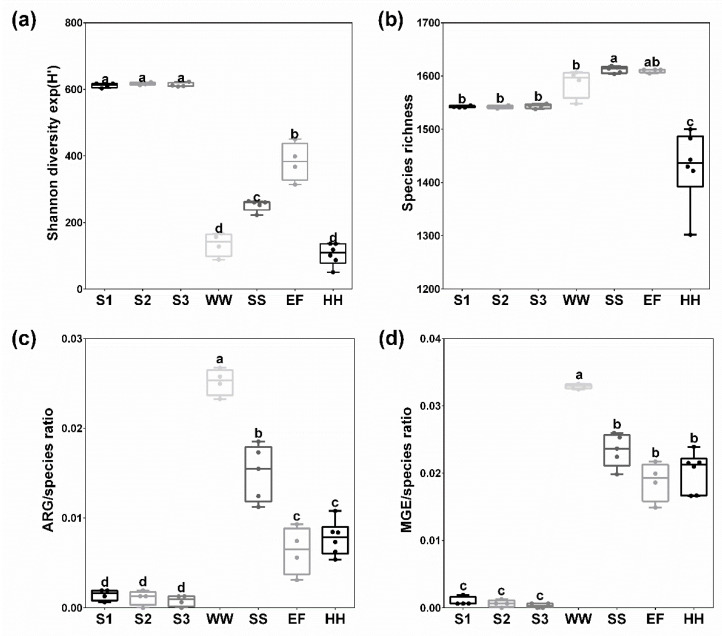
Exponential Shannon index (**a**), species richness (**b**), ARG/species (**c**) and MGE/species ratio (**d**) (count of ARG/MGE per species) of different soils (S1, S2 S3, each *n* = 4), wastewater (WW, *n* = 4), sewage sludge (SS, *n* = 5), WWTP effluent (EF, *n* = 4) and household samples (HH, *n* = 6). Different letters (a–d) indicate significant differences calculated using non-parametric Mann–Whitney test while the same letter above a bar indicates no significant differences. The letters themselves do not have a specific meaning and are only used to ameliorate presentation of significant differences.

The Shannon diversity of the bacterial communities was significantly lower (*p* < 0.05) in WWTP (WW: exp(H′) = 145.4 ± 35.2, SS: exp(H′) = 252.2 ± 17.0 and EF: exp(H′) = 382.9 ± 57.0) and household (HH) samples (exp(H′) = 105.2) compared to soil samples (S1: exp(H′) = 612.2 ± 6.8, S2: exp(H′) = 616.8 ± 3.82 and S3: exp(H′) = 613.4 ± 6.6) (Figure 1a). In contrast, species richness was highest in WWTP samples (*n*(WW) = 1587.3 ± 26.9, *n*(SS) = 1612.0 ± 6.3 and *n*(EF) = 1610.3 ± 3.5) compared to soils (*n*(S1) = 1542.3 ± 1.9, *n*(S2) = 1524.0 ± 2.6 and *n*(S3) = 1543.8 ± 4.2)). Hence, based on the Shannon diversity, species in soil samples were more equally distributed, although the number of different species was highest in WWTP samples. In contrast, a significantly lower species richness and diversity were determined in HH (*n* = 1430 ± 69.8) (Figure 1b). Nearly no ARG (*n*(S1) = 2.25 ± 0.96, *n*(S2) = 1.75 ± 1.26 and *n*(S3) = 1.25 ± 0.96) and MGE (*n*(S1) = 1.50 ± 1.00, *n*(S2) = 1.00 ± 0.82 and *n*(S3) = 0.50 ± 0.58) were detected in soil samples, and thus the ARG/species and MGE/species ratios were significantly higher in WWTP (ARG/species ratio: 0.025 ± 0.001 and MGE/species ratio: 0.033 ± 0.001) and HH samples (ARG/species ratio: 0.008 ± 0.002 and MGE/species ratio:0.02 ± 0.003) (Figure 1c,d). The highest ARG/species and MGE/species ratios occurred in wastewater (WW, ARG/species ratio: 0.025 ± 0.001 and MGE/species ratio: 0.033 ± 0.001) followed by sewage sludge (SS, ARG/species ratio: 0.015 ± 0.003 and MGE/species ratio: 0.023 ± 0.002) while WWTP effluent (EF, ARG/species ratio: 0.006 ± 0.003 and MGE/species ratio: 0.019 ± 0.003) and HH samples revealed a significantly lower ARG/species ratio.

**Figure 2:**
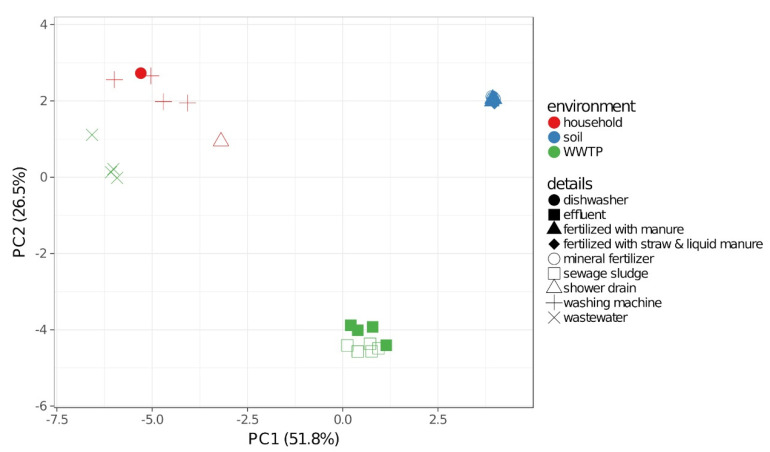
Principal component analysis (PCA) of the beta diversity of different soils, wastewater, sewage sludge, effluent, shower drains, dishwashers and washing machines based on Bray–Curtis dissimilarity. Unit variance scaling was applied to rows and nonlinear iterative partial least squares (NIPALS) PCA was used to calculate principal components. The X- and Y-axis show principal component 1 and principal component 2, which explain 51.8% and 26.5% of the total variance, respectively.

Beta diversity revealed a high degree of variation between the different environments while the samples of the same environment clustered distinctly (Figure 2), revealing that soil samples shared more species with SS (Bray–Curtis index = 0.55) and EF (Bray–Curtis index = 0.53) than with WW (Bray–Curtis index = 0.85) or HH (Bray–Curtis index = 0.75) samples. WW samples clustered closer to HH samples (Bray–Curtis index = 0.73), although Bray–Curtis indices of HH and SS (0.74) were similar or even lower in case of EF samples (0.70). However, SS and EF revealed many similarities (Bray–Curtis index = 0.26), while WW differed strongly from SS (Bray–Curtis index = 0.70) and EF (Bray–Curtis index = 0.72). Moreover, HH samples showed the highest variation within one environment, with an average Bray–Curtis index of 0.55.

### 2.2. Community Composition

**Figure 3:**
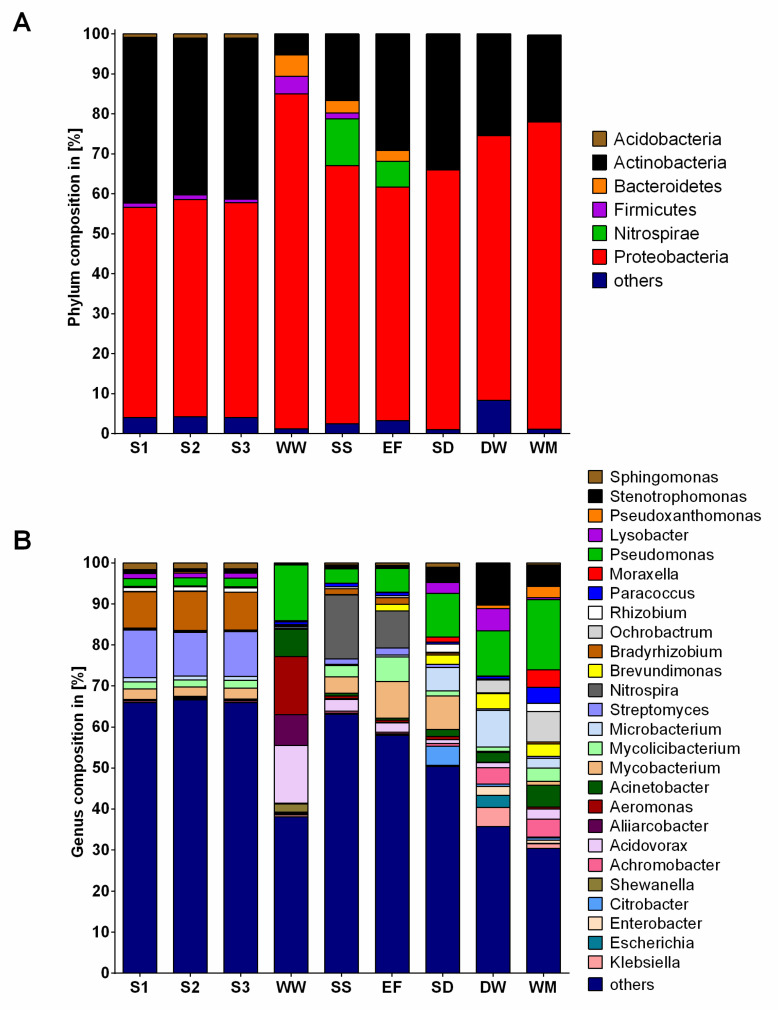
Bacterial composition at phylum (**A**) and genus (**B**) level of different soils (S1, S2, S3, each *n* = 4), wastewater (WW, *n* = 4), sewage sludge (SS, *n* = 4), WWTP effluent (EF, *n* = 4), shower drains (SD, *n* = 1), dishwashers (DW, *n* = 1) and washing machines (WM, *n* = 4) based on next generation sequencing. To ameliorate presentation, only phyla with ≥1% and genera with ≥2% in at least one sample group are shown.

In the present study, we focused on the bacterial community, not considering fungi. The analysis revealed 32 phyla, 670 genera and 1625 species. The majority of identified bacteria belonged to the phylum Proteobacteria (mean: 63.9% ± 10.1%) (Figure 3A). *Pseudomonas* was the only genus to occur in higher fractions across all samples, varying between 1.9% and 17.1% (Figure 3B). Besides the group “others”, *Streptomyces* (11.0% ± 0.5%) and *Bradyrhizobium* (9.2% ± 0.3%) revealed the highest percentages in soil samples. While *Aeromonas* (14.1%) was significantly more prevalent in WW, followed by *Pseudomonas* (13.5%), *Nitrospira* occurred the most in SS and EF samples, with a significantly higher percentage in SS compared to the other environments. The percentage of *Pseudomonas* was significantly higher in washing machine (WM) samples and identified predominantly in all HH samples followed by *Mycobacterium* (8.2%), *Stenotrophomonas* (10.2%) and *Ochrobactrum* (7.4%) in the shower drain (SD), dishwasher (DW) and WM, respectively. Moreover, the highest percentage of *Acinetobacter* was determined in WW and all HH samples (1.7 to 6.7%). Generally, more similarities between WWTP and HH samples were determined compared to soil samples, e.g., regarding the fractions of *Pseudomonas*, *Acidovorax*, *Acinetobacter* or *Brevundimonas*.

### 2.3. Antibiotic Resistance Factors

**Figure 4:**
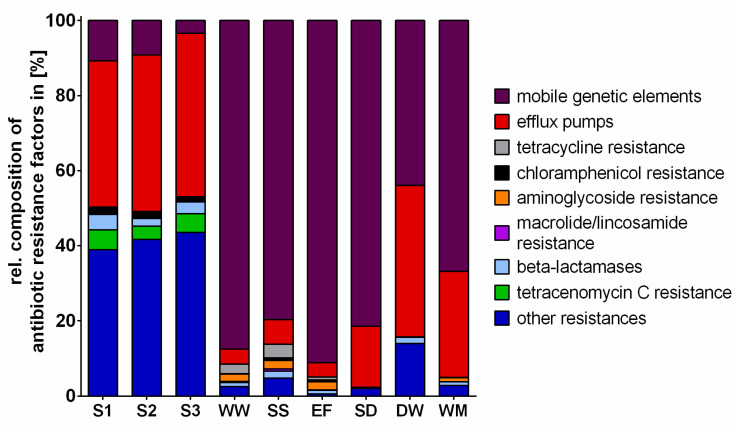
Relative composition of reads annotated to antibiotic resistance factors in soil samples (S1 (*n* = 4), S2 (*n* = 4), S3 (*n* = 4)), WWTP (WW (*n* = 4), SS (*n* = 5), EF (*n* = 4)) and HH samples (SD (*n* = 1), DW (*n* = 1), WM (*n* = 4)). Sequence reads were mapped against the microbial virulence database (MvirDB).

The term “Antibiotic resistance factors” refers to the virulence factor “antibiotic resistance” targeted during the resistance screening of the bioinformatic analysis performed by Eurofins Genomics. These ABR factors include the groups mobile genetic elements (plasmids, transposons, integrons), efflux pumps, tetracycline resistance genes, chloramphenicol resistance genes, aminoglycoside resistance genes, macrolide/lincosamide resistance genes, beta-lactamase genes, tetracenomycin C resistance genes and other resistances, as presented in Figure 4. Compared to WW and HH, the soil samples revealed significantly lower reads, with only approx. 3% accounting for ABR factors (Table S1), while the remaining percentages were annotated to other virulence factors (Figure 4). The majority of the ABR factors were annotated to genes encoding efflux pumps or other resistances, and tetracenomycin C resistance only occurred in soil samples, whereas MGE constituted approx. 7.8% of all ABR factors. In contrast, 41.1% ± 2.9% of ABR factors were determined in WWTP samples and 23.3% ± 7.5% were determined in HH samples, with the lowest percentage found in DW (Table S1). The percentage of MGE, including transposons, integrons and plasmids, was significantly higher (*p* ≤ 0.05) across WWTP samples and represented the main part of the relative composition of ABR factors in HH samples as well. Even though in all HH samples the majority of ABR factors were represented by MGE and efflux pumps, the percentage of MGE in WWTP samples was much higher. Moreover, resistances to aminoglycosides, β-lactams and tetracyclines were more prevalent in WWTP samples.

**Figure 5:**
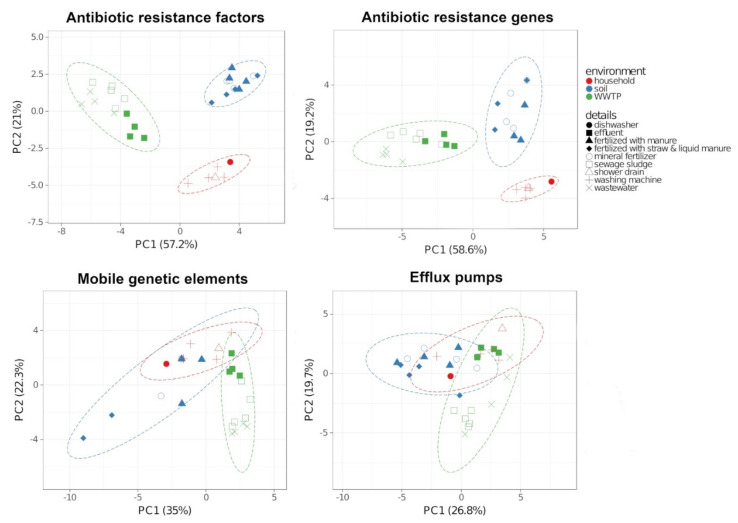
Principal component analysis (PCA) with 95% prediction ellipses based on Spearman correlation of the total of antibiotic resistance factors and its subgroups antibiotic resistance genes, mobile genetic elements and efflux pumps. Unit variance scaling was applied to rows and nonlinear iterative partial least squares (NIPALS) PCA was used to calculate principal components.

Since Spearman correlation is robust to nonlinear relationships and outliers, it was performed prior to principal component analysis (PCA) of ABR factors and ARG, MGE and efflux pumps, which are sub-groups of the ABR factors. Samples of the same environment revealed distinct clustering regarding ABR factors and ARG (Figure 5). Genes encoding ABR factors showed only weak correlations for soil and WWTP samples (r = −0.05), soil and HH (r = 0.02) as well as WWTP and HH samples (r = 0.01). The ARG also correlated weakly in soil and WWTP (r = −0.14), soil and HH (r = −0.03) and WWTP and HH samples (r = −0.16). In contrast, positive correlations of ABR factors in the different sub-environments were determined, revealing the strongest correlations in WWTP samples (r = 0.82), followed by HH samples (r = 0.59), while soil samples showed the weakest correlation (r = 0.18). Regarding MGE, a weak positive correlation for soil and household (r = 0.19), WWTP and household (r = 0.25) and soil and WWTP (r = 0.17) was determined. Inter alia “*Pseudomonas aeruginosa* 2293E multiresistance b-lactamase transposon Tn1412” (Accession no.: L36547.1) was detected in all analyzed environments. In WWTP and household samples, MGEs such as *intI* integrases (AAK50385.1, AAF27722.1, AAO32355.1), transposons (AAS67883.1, AAN61403.1) and plasmids (AAC64416.1, BAB72152.1) as well as tetracycline resistance genes (AAW66497.1) or aminoglycoside resistance genes (YP_001144148.1) were detected within both environments. Moreover, genes encoding efflux pumps positively correlated in soil and WWTP (r = 0.59), soil and HH (r = 0.48) and WWTP and HH (r = 0.44). For all annotated genes, see supplementary material.

**Figure 6:**
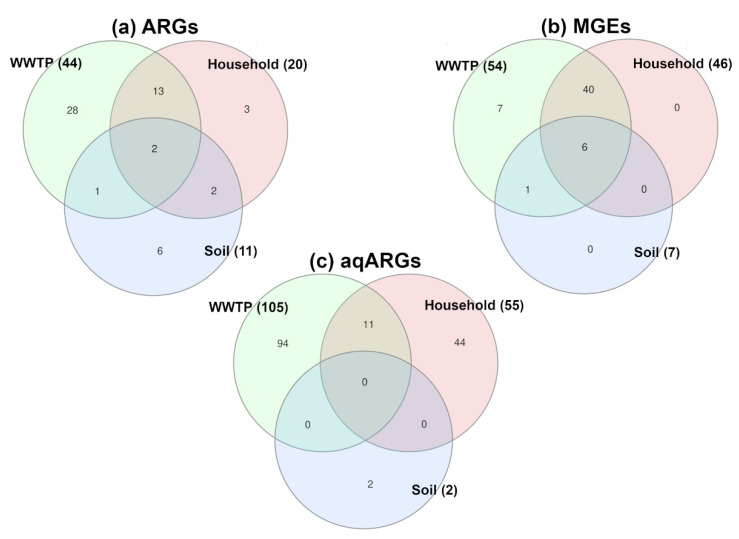
Venn diagram of antibiotic resistance genes (ARG) (**a**) and mobile genetic elements (MGE) (**b**) annotated using MvirDB as well as acquired antibiotic resistance genes (aqARG) (**c**) annotated using Resfinder in the WWTP, soil and HH samples. The number in the bracket indicates the total number of genes annotated for each sample type.

The Venn diagrams created using InteractiVenn [41] of ARG and MGE annotations showed only a small set of shared genes between all three environments, resulting in an overlap of only two (ARG) and six (MGE) genes present in all environments (Figure 6a,b). Most similarities were identified between WWTP and HH samples, with 34.1% of ARG and 85.2% of MGE in WWTP samples present in HH as well. Compared to the similarities between WWTP and HH, soil shared much lower numbers of ARG and MGE with the other investigated environments. However, all MGE annotated in soil and HH samples were determined in WWTP samples. In addition to the screening for virulence factors and ARG using MvirDB, paired end reads were blasted in order to find acquired ARG using ResFinder (Figure 6c, for details see Tables S2–S5 in the supplementary materials), since acquired ARG could be transferred between bacteria. In WWTP and HH samples, an overlap of 11 ARG was determined in these samples, while only two ARG were detected in all soil samples which did not occur in WWTP or HH.

## 3. Discussion

### 3.1. Bacterial Communities and Resistomes in the Different Environments

The results of this cross-environmental study reveal distinct bacterial communities in the different investigated environments, since both alpha and beta diversity of the bacterial microbiome of soil, WWTP and HH samples differed significantly. Moreover, the ABR factors were distinctive for each environment. Studies indicate that the resistome is mainly shaped by bacterial composition [12,13,14], since a shared taxonomy leads to overlaps in MGE and ARG prevalence [15], supporting the distinct clustering of ABR factors and ARG. Hence, our results might indicate that resistances rather seem to develop independently in each environment. This is supported by Munck et al. (2015), determining that less than 10% of the WWTP resistome was found in other environments [42], and Pal et al. (2016), showing clear differences in both ABR pattern and bacterial composition of different environments. Agricultural soils, WWTPs and households are well isolated from each other and have quite different environmental conditions. Hence, differences in the community structure between these environments should be expected. However, small contamination events occurring over time might result in the colonization of the new environment by transferred bacteria (e.g., from HH to WWTP). These varying environmental conditions given in each environment, such as nutrient supply, temperature, pH or wet-and-dry-cycles [43,44,45], might lead to the distinct bacterial compositions and resistomes.

The extremely unique bacterial community and ABR pattern in WWTP might be explained by the beneficial environment consisting of high nutrient levels and a constant temperature, while contaminants such as antibiotic residues, biocides or heavy metals [29,44,46,47] select for antibiotic-resistant bacteria. This confirms previous studies showing a unique bacterial community [40,42] dominated by bacteria originating from the sewer system [40]. WWTP are known to be hotspots for ABR [31,48], which could be supported based on species richness, diversity and proportion of ABR factors as well as ARG and MGE richness in WWTP samples.

The analyzed HH sub-environments have a considerable influx of bacteria originating from humans, animals or foodstuff and are frequently exposed to biocidal, cleaning and personal care products [34,35,49]. Extreme conditions caused by biocides and detergents as well as the frequent change in conditions (e.g., a longer period without use followed by a cleaning cycle in WM and DW) might lead to a selection of well-adapted species and resistant bacteria [43]. This is supported by the low bacterial diversity and significantly higher ARG richness compared to soil samples. Although no comparable data on HH are available so far, studies already determined the prevalence of ABR in households [35,36,37], suggesting that HH might be an under-investigated reservoir of ABR while WWTP and soil are analyzed more frequently. Despite the distinct structure of ABR and the bacterial community, the overlap of identified ARG and MGE in HH and WW highlights that domestic sewage represents an important component of WW. Nonetheless, the ARG richness was significantly lower in HH and the set of shared ARG is most likely connected to the higher diversity and richness of ARG in WWTP samples than environmental transfer, which we did not analyze in our study.

The bacterial community of soil might be comparably less exposed to stressors such as antibiotic residues and industrial pollutants (WWTP) or cleaning and personal care agents (HH). It has to be kept in mind that the soil microbiome is often dominated by fungi in terms of biomass [50,51] and that other microbial groups are frequent, such as protists [52], which were not all detected to a full extent in the present study but surely add to the species richness and diversity of the soil microbiome. Although soil samples revealed the highest taxonomic bacterial diversity and are generally known to harbor a variety of ARG [1,26,53], the analyzed soils revealed by far the lowest percentage of ABR factors. This is further supported by the low ARG and MGE richness determined in soil samples in our and an earlier study [39], indicating that ARG abundance can still be low in diverse microbial communities. However, our findings could also be due to database biases, since the analysis is limited to genes included in MvirDB and Resfinder and generally known ARG, and many ARG occurring in soils were unknown before their first detection [2,25]. Forsberg et al. (2014) determined a low potential for HGT [13] and thus stressors such as antibiotic residues might favor the selection of existing resistant bacteria in soils rather than the acquisition of ARG.

### 3.2. Transfer Between the Environments

HGT between bacteria is especially mediated by MGE such as plasmids, integrons and transposons [17,18], which play a fundamental role in the spread and evolution of ABR [16]. Especially in WWTP, HGT via MGE occurs frequently [54,55,56] and the MGE richness determined in this study was significantly higher compared to soil and HH samples. In contrast, soil samples revealed a low abundance of MGE and a low mobility of the soil resistome has already been determined [13,39], suggesting that HGT of ARG is less likely and transfer is limited compared to WWTP samples [12]. HH and WWTP samples revealed more similarities regarding the genus composition, indicating that some of the same species occur and survive in theset environments. However, we do not know if the sequences of the genes were identical, and hence we cannot provide data proving that the genes were exactly the same. HGT between bacteria requires that they inhabit or at least briefly share the same environment [20], and is induced by substances such as antibiotics [57], which is more likely in case of HH and WWTP samples via the release of domestic sewage. Therefore, the presence of all MGE identified in HH samples in WWTP samples and the overlaps of ARG as well might indicate a dissemination of antibiotic-resistant bacteria from HH to WWTP. However, our study design did not target environmental or genetic transfer, but rather the qualitative analysis of each environment to compare data, allowing assumptions about potential transfers. Since biofilms form frequently in the sampled sub-environments of HH and on sewer pipe surfaces [58,59,60,61] and detected species such as *Pseudomonas* or *Acinetobacter* are especially prone to biofilm formation, this might explain the similarities between WW and HH samples as well. Consequently, to reduce potential transfer of ABR, limiting the development of resistance in the HH sub-environments by reducing the use of antibiotics, antibacterial agents and biocides in the HH or through the occasional use of programs with higher temperatures in WM or DW to remove microbial communities are ways to reduce the spread of ABR.

Although physical forces such as water movement or wind can promote the transfer of soil bacteria [53], dissemination from HH via sewage to WWTP would be more likely, as these environments are immediately interconnected. Antibiotic-resistant strains have been isolated from both the EF and the receiving surface waters downstream of WWTP [47,62,63], highlighting that a transfer to the natural environment is possible as well. However, the application of SS as fertilizer or the irrigation with treated WW is not common in Germany, and hence a transfer from the WWTP to the analyzed soil is highly unlikely. Nevertheless, to decrease the dissemination of ARG from WWTP, the implementation of advanced technologies such as UV treatment or chlorination might minimize possible risks [63,64]. In contrast, all ARG and MGE in SS and EF were shared with WW, while nearly all genes detected in SD and DW matched the WM samples (Figure S1). This further supports the hypothesis of ABR development or gene transfer within the same environment dominating, compared to the exchange between environments. While many factors need to be fulfilled for a transfer between different environments [65], a transfer between bacteria within the same environment can occur frequently. Furthermore, ABR can develop due to spontaneous mutations promoted by selective pressure of antibiotics or biocides [66], which might contribute to the distinct resistomes of the analyzed environments as well. Even though these environments can still act as reservoirs for ABR, since many ARG detected in pathogens seem to have their origin in environmental bacteria [11,67,68,69], we assume that the transfer between distinct environments might occur less frequently than expected. However, in-depth studies are necessary to support or prove our assumptions made within this study.

## 4. Materials and Methods

### 4.1. Sample Collection and Preparation

Samples of the inner tubing of shower drains (SD), dishwasher sumps and sieves (DW), detergent trays and rubber door seals of washing machines (WM) were taken from households in the vicinity of Kleve, Germany, between September 2018 and June 2019 in a previous study [37]. All samples were taken in households from different apartment buildings or single-family houses. The surfaces of the inner tubing of SD, the sumps and sieves of DW or the detergent trays and rubber door seals of WM were sampled using a sterile cotton swab in triplicate.

Wastewater (WW), sewage sludge (SS) and effluent (EF) were collected in another study [10] at a secondary wastewater treatment plant (WWTP) in the district of Kleve each week over a one-year period (May 2018 to April 2019). In this WWTP, a total of approximately 5.5 million m³ WW is treated every year, resulting in 7.300 t of SS. The influent comprises approx. 53,035 households, WW from industries (5% of the WW) and the area includes three different clinics with a total of 1.280 beds. Samples were prepared as described in the previous studies [10,37], since the same samples were analyzed before using qPCR and a microbiological approach.

Soil samples were taken in 2019 from a long-term experiment (since 2001) in the district of Kleve with different fertilization treatments aiming at evaluating the effects on potato, yield and quality. Three treatments were selected for next-generation sequencing: treatment 1 (S1) was fertilized with mineral fertilizer, treatment 2 (S2) was fertilized with cattle manure and treatment 3 (S3) with straw and liquid cattle manure. The different treatments were chosen to represent a soil without direct impact of manure and two with the impact of different types of manure, since studies showed an increase in ARG abundance in soil after manure application [5,70]. Each different treatment was represented in four plots of 9 m × 9 m and five sub-samples were taken in each plot at 30 cm depth and pooled. Samples were then sieved to 2 mm prior to DNA extraction.

### 4.2. DNA Extraction

For purification of total DNA, the Fast DNA Spin Kit for Soil (MP Bio, Santa Ana, CA, USA) was used according to the manufacturer’s instructions with the following adjustments:

In the case of domestic samples (SD, DW and WM), instead of 500 mg of solid material, 250 µL of a suspended sample was applied to the lysing matrix tube. Samples were homogenized twice in the FastPrep-24™ instrument for 60 s at 6.0 m s^−1^ [37]. WW, SS and EF samples were each pooled from one month and WW and SS samples were centrifuged at 4800 g/8 °C/10 min. While the pellets of WW samples were re-suspended in 200 mL of sterile 0.9% sodium chloride, SS samples were washed with 10 mL of sterile 0.9% sodium chloride twice. Samples were centrifuged and re-suspended in sodium chloride again and 500 μL of the sample was added to the lysing matrix tubes. Each 500 µL sample was added to the lysing matrix tubes and WW samples were homogenized as the household samples and SS samples three times for 40 s at 5.0 m s^−1^ in the FastPrep-24™ instrument. EF samples were filtered through a 0.2 μm pore size filter which was added in small pieces to the lysing matrix tubes followed by homogenization for 60 s at 6.0 m s^−1^ [10]. DNA from soil samples (S1, S2, S3) was extracted with the following modifications [71]: 950 and 120 mL of sodium phosphate buffer and “MT buffer” were used, respectively. Samples were homogenized twice in FastPrep-24™ instrument for 45 s at a 6.5 m s^-1^. After that, samples were centrifuged at 14,000 g/5 min at room temperature. To remove contaminants, the DNA bound to the binding matrix of the kit was washed twice with 1 mL of 5.5 M guanidine thiocyanate (Carl Roth, Karlsruhe, Germany).

### 4.3. Metagenomic Analysis 

Since the study aimed to analyze the microbial communities of different environments, the INVIEW Metagenome analysis from Eurofins Genomics (Eurofins Genomics Germany GmbH, Ebersberg, Germany) was chosen for an NGS-based taxonomic, resistance and functional profiling of the samples. All household, WW, SS and EF samples of the previous studies were divided into groups based on DNA concentration and sample pools were prepared. These samples were chosen because they were taken in the same geographical area. The pools consisted of two individual samples each in the case of WW, SS and EF samples, while the household sample pools comprised five (DW), seven (SD), nine (pool WM1-WM3) and five (pool WM4) individual samples. Sample numbers varied because samples with too low concentrations of DNA were excluded. The soil samples were also taken within the same geographical area and pools of each four samples of the same treatment were prepared. Samples were pooled and DNA quantity was properly balanced in each pool to equally represent each genome. To achieve this, the DNA concentration of the individual samples was adjusted to the same concentration and equal amounts of each sample were pooled. Sequencing and library preparation were performed at Eurofins Genomics using Genome Sequencer Illumina Hiseq platform with the NovaSeq 6000 S2 PE150 XP system. Bioinformatic analysis was performed by Eurofins Genomics including taxonomic profiling and screening for virulence factors (antibiotic resistance, differential gene regulation, pathogenicity islands, protein toxins, transcription factors and virulence protein) using MvirDB. In addition, paired end reads were blasted in order to identify acquired ARG using ResFinder [72], which is not differentiated in MvirDB. A total of 870,593,530 high-quality sequences were acquired from the pooled DNA samples of soils (S1 (*n* = 4), S2 (*n* = 4), S3 (*n* = 4)), WWTP (WW (*n* = 4), SS (*n* = 5), EF (*n* = 4)) and households (*n* = 6, each one comprising the DNA pool of SD and DW samples and 4 DNA pools of WM samples). For details see Tables S6 and S7 in the supplementary materials.

### 4.4. Data Analysis

Statistics were performed using GraphPad Prism (GraphPad Software Inc.). Data were expressed as means (± standard error). Alpha diversity (Shannon diversity exp(H′)), the number of ARG and MGE, was not normally distributed, and thus the Mann–Whitney test (*p* ≤ 0.05) was performed to identify significant differences between the environments. The structure of the microbial community was compared using a principal component analysis (PCA) based on Bray–Curtis dissimilarity of the different environments using ClustVis [73]. The bacterial communities and ABR factor compositions were compared using the non-parametric Mann–Whitney test (*p* ≤ 0.05). We explored the underlying relationships between antibiotic resistance factors, ARG, MGE and efflux pumps using PCA based on a Spearman correlation, since it is robust to nonlinear relationships and outliers.

## 5. Conclusions

We observed distinctive resistomes and only a few overlaps of ARG between the targeted environments, suggesting that ABR might predominantly develop in individual environments as caused by the distinct environmental conditions. A transfer of ABR between the different environments is more limited to directly connected environments (e.g., HH to WWTP). The investigated environments revealed distinct bacterial communities with pronounced differences between sub-environments in WWTP and HH, supporting the hypothesis that resistomes are predominantly structured by bacterial phylogeny. Hence, despite its limitations, our study provides evidence that the transfer of ARG and antibiotic-resistant bacteria between different environments might be less important than focusing on the implementation of prevention measures in each individual environment. While our study gives an overview only allowing assumptions, further studies comprising more samples and in-depth analysis of shared genes are needed. ABR spread can be limited by reducing the development of resistance in the HH environment, to prevent a low but still possible infection risk for household members and a dissemination within the HH or via domestic sewage to the WWTP. Moreover, a possible transfer of antibiotic residues and resistant bacteria from WWTP to terrestrial and aquatic environments should be avoided to prevent the promotion of resistance development. This could be achieved by implementing advanced technologies in WW treatment and the prevention of the use of treated WW or SS for fertilization/irrigation. However, more comprehensive studies are needed to compare the resistomes of different environments and to confirm whether or not ABR develops independently in each environment, since our study did not target ARG transfer. With this qualitative analysis, we aimed to provide first insights to support our hypothesis that ABR develops and clusters in each distinct environment. Based on our data, quantitative analyses should be performed to further elaborate the significance of the differences between the analyzed environments.

## Data Availability

The metagenome data presented in this study are openly available at http://www.ebi.ac.uk/ena/data/view/PRJEB41538.

## Supplementary Materials

The following are available online at https://www.mdpi.com/article/10.3390/antibiotics10050514/s1, Figure S1: Venn diagram of antibiotic resistance genes and mobile genetic elements in WWTP (A, C) and household samples (B, D) annotated using MvirDB. The number in the bracket indicates the total number of genes annotated for each sample type. Table S1: Percentage and number of reads of virulence factor annotated to ABR factors, Table S2: Acquired antibiotic resistance genes with accession number identified via Resfinder in Wastewater (WW) samples, Table S3: Acquired antibiotic resistance genes with accession number identified via Resfinder in sewage sludge (SS) samples, Table S4: Acquired antibiotic resistance genes with accession number identified via Resfinder in WWTP effluent (EF) and soil (2A and 3C) samples, Table S5: Acquired antibiotic resistance genes with accession number identified via Resfinder in dishwasher (DW), shower drain (SD) and washing machine (W) samples, Table S6: Sequence reads per samples of soil (S1, S2, S3), WWTP (WW, SS, EF) and household samples (SD, DW, WM) and Table S7: Percentage of reads assigned to kingdoms in different soils (S1, S2, S3), wastewater (WW), sewage sludge (SS), effluent (EF), shower drains (SD), dishwashers (DW) and washing machines (WM) in percent.
