# Supplementary material for: Distinct Resistomes and Microbial Communities of Soils, Wastewater Treatment Plants and Households Suggest Development of Antibiotic Resistances Due to Distinct Environmental Conditions in Each Environment
Source: Antibiotics (Basel). 2021 May 1;10(5):514. doi: 10.3390/antibiotics10050514 (PMC8147267; doi:10.3390/antibiotics10050514)
Supplement: Supplementary file 1 [file antibiotics-10-00514-s001.zip › antibiotics-1172837-supplementary.pdf]

Supplementary data

Reads assigned to antibiotic resistance factors

Table S1: Percentage and number of reads of virulence factor annotated to ABR factors.

sample	percentage of ABR factors	number of reads
**S1**	3.2%	20
**S2**	3.1%	18
**S3**	3.4%	18
**WW**	40.7%	8132
**SS**	44.1%	1150
**EF**	38.4%	584
**SD**	28.2%	6002
**DW**	14.6%	6532
**WM**	27.1%	4971

Acquired antibiotic resistance genes (Resfinder)

Table S2: Acquired antibiotic resistance genes with accession number identified via Resfinder in Wastewater (WW) samples.

**WW1**	**Accession no.**	**WW3**	**Accession no.**	**WW4**	**Accession no.**	**WW5**	**Accession no.**
aadA10	U37105	aadA11	AY144590	aadA11	AY144590	aadA11	AJ567827
aadA11	AJ567827	aadA13	AY713504	aadA13	AY713504	aadA11	AY144590
aadA11	AY144590	aadA2	NC_010870	aadA5	AF137361	aadA13	AY713504
aadA16	EU675686	aadA5	AF137361	ant(3")-Ia	X02340	aadA16	EU675686
aaDA5	AF137361	aadA6	AF140629	aph(3")-Ib	AF024602	aadA5	AF137361
aadA6	AF140629	ant(3")-Ia	X02340	aph(3')-Ia	V00359	ant(2")-Ia	X04555
ant(3´´)-Ia	X02340	aph(3")-Ib	AF024602	aph(6)-Id	M28829	ant(3")-Ia	02340
ant(6)-Ia	KF421157	aph(6)-Id	M28829	blaAER-1	U14748	ant(6)-Ia	KF421157
ant(6)-Ia	KF864551	blaAER-1	U14748	blaCARB-10	EU850412	ant(6)-Ia	KF864551
aph(3´´)-Ib	AF024602	blaGES-5	DQ236171	blaGES-5	DQ236171	aph(3")-Ib	AF024602
aph(6)-Id	M28829	blaNPS	AY027589	blaLCR-1	X56809	aph(3")-Ib	AF321551
blaAER-1	U14748	blaOXA-10	J03427	blaOXA-1	HQ170510	aph(6)-Id	M28829
blaLCR-1	X56809	blaOXA-119	DQ767903	blaOXA-10	J3427	blaAER-1	U14748
blaNPS	AY027589	blaOXA-2	DQ112222	blaOXA-119	DQ767903	blaBEL-1	DQ089809
blaOXA-10	J03427	blaOXA-20	AF024602	blaOXA-129	FJWZ01000025	blaCARB-10	EU850412

blaOXA-101	AM412777	blaOXA-205	JF800667	blaOXA-2	DQ112222	blaGES-5	DQ236171
blaOXA-119	DQ767903	blaOXA-347	ACWG01000053	blaOXA-205	JF800667	blaLCR-1	X56809
blaOXA-129	FJWZ01000025	blaOXA-392	AB901044	blaOXA-211	JN861779	blaNPS	AY027589
blaOXA-2	DQ112222	blaOXA-427	KX827604	blaOXA-296	APOH01000009	blaOXA-10	J03427
blaOXA-205	JF800667	blaVEB-1	HM370393	blaOXA-333	KF203107	blaOXA-101	AM412777
blaOXA-211	JN861779	cfxA3	AF472622	blaOXA-347	ACWG01000053	blaOXA-119	DQ767903
blaOXA-296	APOH01000009	mcr-3.6	MF598076	blaOXA-392	AB901044	blaOXA-129	FJWZ01000025
blaOXA-347	ACWG01000053	ere(D)	KP265721	blaOXA-427	KX827604	blaOXA-2	DQ112222
blaOXA-392	AB901044	erm(B)	U86375	blaVEB-1	DQ393569	blaOXA-20	AF024602
blaOXA-4	AY162283	erm(F)	M17808	cfxA3	AF472622	blaOXA-205	JF800667
blaOXA-427	KX827604	lnu(B)	JQ861959	cfxA6	GQ342996	blaOXA-211	JN861779
blaOXA-58 A	Y665723	lnu(C)	AY928180	ere(D)	KP265721	blaOXA-296	APOH01000009
blaVEB-1	HM370393	lnu(F)	EU118119	erm(B)	U86375	blaOXA-333	KF203107
cfxA3	AF472622	lsa(E)	JX560992	erm(F)	M17808	blaOXA-347	ACWG01000053
mcr-5.1	KY807921	mef(A)	AF227520	lnu(B)	JQ861959	blaOXA-35	AF315786
ere(A)	AF099140	mef(A)	AJ971089	lnu(C)	AY928180	blaOXA-392	AB901044
ere(A)	DQ157752	mef(A)	HG423652	lnu(F)	EU118119	blaOXA-4	AY162283
ere(D)	KP265721	mef©	AB571865	lnu(G)	KX470419	blaOXA-427	KX827604
erm(47)	KU612222	mph(A)	D16251	lsa(E)	JX560992	blaOXA-58	AY665723
erm(B)	U86375	mph E	DQ839391	mef(A)	HG423652	blaVEB-1	HM370393
erm(F)	M17808	mph(G)	AB571865	mef(A)	U83667	cfxA5	AY769934
lnu(B)	JQ861959	msr(D)	AF227520	mef(B)	FJ196385	ere(A)	AF099140
lnu(F)	AJ561197	msr(E)	FR751518	mef(C)	AB571865	ere(D)	KP265721
lnu(F)	EU118119	aac(6')-Ib-cr	DQ303918	mph(A)	D16251	erm(47)	KU612222
lsa(E)	JX560992	qnrS2	DQ485530	mph(E)	DQ839391	erm(B)	U86375
mef(A)	AF227520	sul1	U12338	mph(G)	AB571865	erm(F)	M17808
mef(A)	HG423652	sul2	AY034138	mph(N)	KF648874	lnu(B)	JQ861959
mef(B)	FJ196385	tet(39)	KT346360	msr(D)	AF227520	lnu(C)	AY928180
mef©	AB571865	tet(A)	AF534183	msr(D)	AF274302	lnu(F)	EU118119
mph(E)	DQ839391	tet(C)	AF055345	msr(E)	FR751518	lnu(G)	KX470419
mph(G)	AB571865	tet(E)	CP000645	aac(6')-Ib-cr	DQ303918	lsa(E)	JX560992
msr(D)	AF227520	tet(Q)	L33696	qnrS2	DQ485530	mef(A)	AF227520
msr(D)	AF274302	tet(Q)	X58717	qnrVC4	GQ891757	mef(A)	AJ971089
msr(E)	FR751518	tet(W)	AJ427422	sul1	U12338	mef(A)	HG423652
catB3	U13880	dfrA14	KF921535	sul2	AY034138	mef(B)	FJ196385

cmlA1	M64556			tet(39)	KT346360	mef(C)	AB571865
cmx	U85507			tet(A)	AJ517790	mph(A)	D16251
aac(6')-Ib-cr	DQ303918			tet(C)	AY046276	mph(E)	DQ839391
qnrS2	DQ485530			tet(E)	CP000645	mph(G)	AB571865
sul1	AY963803			tet(M)	X04388	msr(D)	AF227520
sul2	AY034138			tet(Q)	L33696	msr(D)	AF274302
tet(36)	AJ514254			tet(Q)	X58717	msr(E)	FR751518
tet(39)	KT346360			tet(W)	AJ427422	catB3	U13880
tet(A)	AJ517790			tet(X)	M37699	catQ	M55620
tet(C)	AY046276			dfrA12	AM040708	cmlA1	M64556
tet(E)	CP000645			dfrA14	DQ388123	cmx	U85507
tet(M)	X04388					aac(6')-Ib-cr	DQ303918
tet(Q)	L33696					qnrS2	DQ485530
tet(W)	AJ427422					qnrVC4	GQ891757
dfrA14	KF921535					sul1	AY963803
						sul2	AY034138
						tet(39)	KT346360
						tet(40)	FJ158002
						tet(A)	AF534183
						tet(C)	AF055345
						tet(E)	CP000645
						tet(M)	X04388
						tet(O/32/O)	FP929050
						tet(Q)	L33696
						tet(Q)	X58717
						tet(Q)	Z21523
						tet(W)	AJ427422
						tet(X)	M37699

Table S3: Acquired antibiotic resistance genes with accession number identified via Resfinder in sewage sludge (SS) samples.

**SS1**	**Accession no.**	**SS2**	**Accession no.**	**SS3**	**Accession no.**
ant(6)-Ia	KF421157	aadA11	AJ567827	aadA2	NC_010870
blaOXA-296	APOH01000009	ant(6)-I	FN594949	mph(A)	U36578
blaOXA-5	AF347074	erm(47)	KU612222	sul1	AY963803
erm(B)	U86375	erm(B)	X66468		
lnu(B)	JQ861959	lsa(E)	JX560992		
lsa(E)	JX560992	mef(A)	HG423652		
mef(A)	HG423652	mef(B)	FJ196385		
mph(E)	DQ839391	msr(D)	AF227520		
mph(N)	KF648874	msr(D)	AF274302		
msr(D)	AF227520	msr(E)	FR751518		
msr(D)	AF274302	sul1	AY963803		
msr(E)	FR751518	sul2	AY034138		

tet(M)	AM990992	catQ	M55620
sul1	AY963803	tet(W)	AJ427422
catQ	M55620		

**SS4**	**Accession no.**	**SS5**	**Accession no.**
blaOXA-10	J03427	aph(6)-Id	M28829
blaOXA-129	FJWZ01000025	blaOXA-5	AF347074
lnu(B)	JQ861959	erm(47)	KU612222
sul1	AY963803	erm(B)	U86375
		erm(F)	M17808
		lnu(B)	JQ861959
		lsa€	JX560992
		mef(A)	HG423652
		mph(E)	DQ839391
		msr(D)	AF227520
		msr(D)	AF274302
		msr(E)	FR751518
		sul1	AY963803
		sul2	AY034138
		EstDL136	JN242251
		catQ	M55620

Table S4: Acquired antibiotic resistance genes with accession number identified via Resfinder in WWTP effluent (EF) and soil (2A and 3C) samples.

**EF1**	**Accession no.**	**EF3**	**Accession no.**	**S2-1**	**Accession no.**
sul1	AY963803	aph(3")-Ib	AF024602	dfrB3	FM877478
		erm(B)	AF299292		
		sul1	U12338		
		cmx	U85507		
**EF4**	**Accession no.**	**EF5**	**Accession no.**	**S3-3**	**Accession no.**
aadA11	AJ567827	blaOXA-20	AF024602	dfrB3	X72585
sul1	U12338	sul2	AY034138		

Table S5: Acquired antibiotic resistance genes with accession number identified via Resfinder in dishwasher (DW), shower drain (SD) and washing machine (W) samples.

**DW**	**Accession no.**	**SD**	**Accession no.**	**WM1-4**	**Accession no.**
aac(6')-Iih	AJ584701	aph(3")-Ib	AF024602	aac(3)-Ich	CP000490
aac(6')-Iz	AF140221	aph(3')-IIb	CP006832	aadA10	U37105
aph(3')-Iic	AM743169	aph(6)-Id	M28829	aadA11	AY144590
blaACT-14	JX440354	blaCMY-49	GQ402541	aph(3")-Ib	AF321551
blaADC-25	EF016355	blaOXA-50	AY306130	aph(6)-Id	M28829
blaEBR-1	AF416700	fosA	ACWU01000146	blaACT-12	JX440355
blaOCH-2	AJ295340	catB7	AF036933	blaCMY-25	EU515249

blaOCH-7	AJ295345	crpP	HM560971	blaOCH-7	AJ295345
blaOXA-258	HE614014	qnrB1	DQ351241	blaOXA-373	HG931732
blaOXA-417	KM220587			blaSHV-187	LN515533
blaOXY-5-1	AJ871868			blaSHV-75	AM176550
fosA	M85195			fosA	ACWO010000 79
mdf(A)	Y08743			oqxA	EU370913
tet(42)	EU523697			oqxB	EU370913
				sul1	AY963803
				sul2	AY034138
				aadA10	U37105
				ant(3")-Ia	X02340
				aph(3")-Ib	AF024602
				aph(3')-Iic	AM743169
				aph(6)-Id	M28829
				blaHERA-1	AF311385
				blaOCH-4	AJ295342
				blaOXA-114c	HM056041
				blaOXA-373	HG931732
				catA1	V00622
				crpP	HM560971
				cat(pC194)	NC_002013
				catB7	AF036933
				fosA	ACWU010001 46
				aadA10	U37105
				aph(3')-Iib	CP006832
				aph(6)-Id	M28829
				blaOXA-114c	HM056041
				blaOXA-134	HQ122933
				blaOXA-212	JN861780
				blaOXA-282	APQS0100001 9
				blaOXA-283	AYHO0100000 5
				blaOXA-333	KF203107
				blaOXA-488	CP017969
				blaPAO	AY083592
				aadA6	AF140629
				aph(3")-Ib	AF024602
				aph(3')-IIb	CP006832
				aph(6)-Id	M28829
				blaOCH-5	AJ295343
				blaOCH-6	AJ295344
				blaOCH-7	AJ295345
				blaOXA-114c	HM056041
				blaOXA-212	JN861780
				blaOXA-396	AY306134
				blaPAO	FJ666073
				crpP	HM560971

	sul1	U12338
	sul2	AY034138

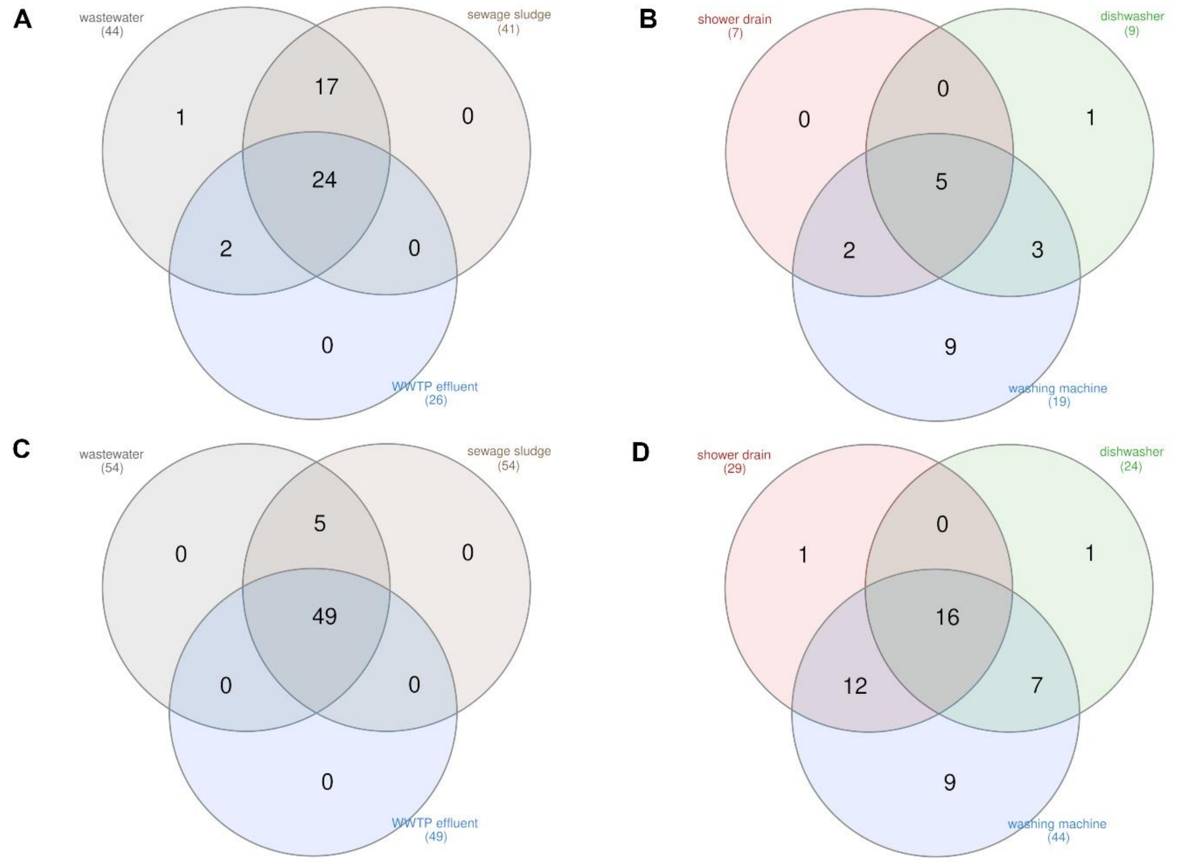

Figure S1: Venn diagram of antibiotic resistance genes and mobile genetic elements in WWTP (**A**, **C**) and household samples (**B**, **D**) annotated using MvirDB. The number in the bracket indicates the total number of genes annotated for each sample type.

Sequencing reads

Table S6: Sequence reads per samples of soil (S1, S2, S3), WWTP (WW, SS, EF) and household samples (SD, DW, WM).

sample	total reads	high quality reads	sample	total reads	high quality reads
**S1.1**	30,155,842	29,392,172	**SS1**	27,841,748	27,184,980
**S1.2**	30,252,856	29,487,760	**SS2**	29,552,232	28,842,838
**S1.3**	33,207,516	32,049,660	**SS3**	23,844,194	23,306,058
**S1.4**	27,088,328	26,289,070	**SS4**	28,851,940	28,162,328
**S2.1**	27,874,140	27,145,314	**SS5**	45,557,500	44,544,338
**S2.2**	22,138,400	21,476,644	**EF1**	29,956,520	29,260,546

**S2.3**	36,275,070	35,326,374	**EF3**	27,685,258	27,026,562
**S2.4**	26,881,178	26,241,796	**EF4**	29,199,610	28,493,828
**S3.1**	25,784,402	25,198,700	**EF5**	30,428,190	29,768,104
**S3.2**	28,240,628	27,420,090	**SD5**	31,657,982	30,737,836
**S3.3**	25,678,742	24,967,918	**DW**	24,271,104	23,617,480
**S3.4**	22,515,406	21,833,202	**WM2**	32,182,966	31,423,310
**WW1**	31,810,164	30,987,066	**WM3**	25,906,062	25,300,232
**WW3**	25,286,020	24,730,394	**WM4**	27,960,538	27,110,180
**WW3**	23,253,200	22,800,878	**WM5**	29,590,952	28,754,122
**WW5**	32,467,628	31,713,750			

Taxonomic distribution

Table S7: Percentage of reads assigned to kingdoms in different soils (S1, S2, S3), wastewater (WW), sewage sludge (SS), effluent (EF), shower drains (SD), dishwashers (DW) and washing machines (WM) in percent.

	S1	S2	S3	WW	SS	EF	SD	DW	WM
**Archaea**	0.4	0.5	0.4	0.2	0.3	0.1	0.0	0.0	0.0
**Bacteria**	97.4	97.3	97.4	99.3	98.1	96.5	99.6	97.7	99.5
**Eukaryota**	0.5	0.5	0.5	0.1	0.4	1.6	0.1	0.0	0.1
**Fungi**	0.5	0.4	0.5	0.1	0.3	0.6	0.1	1.9	0.1
**Viruses**	0.0	0.0	0.0	0.1	0.0	0.1	0.0	0.1	0.1
**Ambiguous**	1.2	1.3	1.2	0.3	0.9	1.2	0.2	0.3	0.2
